# Stability of Carotenoid Diets During Feed Processing and Under Different Storage Conditions

**DOI:** 10.3390/molecules17055651

**Published:** 2012-05-11

**Authors:** Orapint Jintasataporn, Bundit Yuangsoi

**Affiliations:** 1 Department of Aquaculture, Faculty of Fisheries, Kasetsart University, Bangkok 10900, Thailand; 2 Department of Fisheries, Faculty of Agriculture, Khon Kaen University, Khon Kaen 40002, Thailand

**Keywords:** carotenoid, stability, feed processing, storage

## Abstract

The stability of formulated carotenoid diets during feed processing and under different storage conditions were studied. All carotenoid diets were split into two groups with one group containing BHT (acting as an antioxidant) at 250 ppm and the other without BHT. The experiment was divided into two parts. First, all diets were evaluated in total carotenoid (TC) loss during feed processing, in dry mixed feeds after being processed and dried. In the final part, the completed dietary carotenoids were stored in an aluminum foil bag, the top of which was sealed with a bag sealer and kept under different storage conditions at 26–28 °C and 4 °C. The stability of the TC was observed during an 8-week trial period. The results showed that the diet pelleting process did not affect the carotenoid content of the diets, and the best storage temperature for the formulated carotenoid diet was at 4 °C. However, an antioxidant was added to assist in energy saving before feed processing. Thus, the addition of BHT at 250 ppm can be done at normal room temperature in order to reduce oxidation that might cause a loss of TC quantities in diets.

## 1. Introduction

Carotenoids, which are lipid soluble pigments, are responsible for the skin color of ornamental fish. The yellow, orange and red hues found in fish skin are the result of a group of carotenoid pigments [1]. Carotenoids are also vital nutrients for healthy growth, metabolism and reproduction. Since fish, like other animals, are not able to synthesize carotenoids, they have to obtain them from dietary sources [2,3,4]. Different species have different metabolic characteristics for different carotenoid species, but they can modify alimentary carotenoids and store them in the integument and other tissues [2]. The composition of carotenoids differs qualitatively and quantitatively between diets. It also varies in a given diet due to factors, such as processing and storage conditions [5]. Micronutrients, such as carotenoids and vitamins, are more likely to be damaged by the feed manufacturing process [6]. Vitamins and carotenoids are sensitive organic compounds that can be denatured by water, oxygen, trace minerals, heat and other factors [7,8]. The feed process involves water, heat, and mechanical stresses, all of which can impact on vitamin and carotenoid stability [5]. Feed processing is a versatile high temperature process that may alter the nutritional value of feeds [9]. 

Today, carotenoids are often measured by HPLC, allowing for individual carotenoid detection and quantification, with the disadvantage of being more time and cost intensive. Spectrophotometric measurements are widely used and have been adapted for special purposes, that is, the analysis of food items rich in specific carotenoid species employing selected wavelengths and their corresponding absorption coefficients. A comparison of spectrophotometric measurements to determine total carotenoid content compared to HPLC in various fruits and vegetables resulting in a close estimation of total carotenoid content compared to HPLC including a simple, rapid, and cost-effective method to quantify total carotenoids [10,11].

Synthetic antioxidants are used as preservatives in fish feed and fish feed ingredients to stop or at least slow down auto-oxidation. It is recommended that antioxidants which are preventive and curative, for example ethoxyquin (EQ), butylated hydroxytoluene (BHT), butylated hydroxyanisole (BHA), octylgallate and propylgallate be generally added to some raw materials used in fish feed and they are almost always added during feed manufacture. Hwang *et al*. [12] reported that the major antioxidant in fish meal and fish feed in Taiwan was BHT and levels were up to 150 mg kg^−1^. Yuangsoi *et al*. [13] indicated that supplemental BHT at 250 ppm in tilapia diet effectively retarded rancidity. Among the factors listed above, mainly heat and the presence of oxygen were anticipated to influence carotenoid stability during the production of dry feed. However, processing and storage of diet should, be optimized to prevent carotenoid loss [5]. Thus, it is important to learn about the stability of dietary carotenoids. The objectives of this study were to evaluate stability of dietary carotenoids during pelletizing processing and to investigate the stability of carotenoids in formulated carotenoid diets under different storage conditions.

## 2. Results and Discussion

Investigation of the stability of formulated carotenoid diets were conducted in two parts. First, all diets were evaluated for total carotenoid (TC) loss during feed processing. In the final part, the dietary carotenoids were stored in an aluminum foil bag, the top of which was sealed with a bag sealer and kept at different storage conditions at 26–28 °C and 4 °C during an 8-week trial period. 

### 2.1. Stability of Carotenoid Diets during Feed Processing

Dietary carotenoids were divided into two subgroups, with and without antioxidant. The stability of total carotenoid content (TC) in the diets being tested during the three production processes is shown in Table 1. The data shows that there was no interaction between dietary carotenoids and unit operations (*p* ≥ 0.05). This indicates that TC quantities were quite stable in both subgroups from the beginning of the production process until the processing of dried feed (as shown in Table 2).

**Table 1:** Total carotenoids content in the experimental diets at the various unit operations.

Experimental Diets	Total carotenoid (mg kg^−1^)	
	Without antioxidant	With antioxidant
**Dry mix**		
Diet 1	5.72 ± 0.12	5.99 ± 0.55
Diet 2	27.44 ± 4.89	27.64 ± 0.18
Diet 3	27.78 ± 1.84	28.49 ± 1.12
Diet 4	26.74 ± 4.65	28.57 ± 3.55
Diet 5	47.89 ± 2.93	48.51 ± 3.93
Diet 6	88.49 ± 7.95	87.27 ± 2.99
**After being processed**		
Diet 1	5.60 ± 0.10	5.86 ± 0.19
Diet 2	26.60 ± 0.54	27.00 ± 2.63
Diet 3	25.26 ± 1.87	27.08 ± 1.59
Diet 4	25.34 ± 3.39	27.98 ± 1.50
Diet 5	46.68 ± 1.88	47.16 ± 2.01
Diet 6	86.13 ± 2.25	86.17 ± 3.76
**Drying**		
Diet 1	5.54 ± 0.19	5.70 ± 0.27
Diet 2	25.56 ± 0.98	26.91 ± 0.84
Diet 3	23.67 ± 2.45	26.08 ± 0.75
Diet 4	25.04 ± 2.97	26.25 ± 2.90
Diet 5	45.85 ± 4.72	46.75 ± 3.08
Diet 6	84.97 ± 4.28	86.01 ± 3.00
*P*-value	0.0001	0.0001
Diet	0.0001	0.0001
Unit operations	0.1417	0.1770
Diet*Unit operations	0.9989	0.9991

**Table 2:** Comparing total carotenoids content in the experimental diets between two subgroups which without and with antioxidant at the various unit operations.

Experimental diets	Total carotenoid (mg kg^−1^)		
	Without antioxidant	With antioxidant	*P*-value
Diet 1	5.62 ± 0.14	5.85 ± 0.34	0.57
Diet 2	26.54 ± 2.14	27.18 ± 1.21	0.30
Diet 3	25.57 ± 2.06	27.21 ± 1.15	0.51
Diet 4	25.71 ± 3.68	27.60 ± 2.65	0.72
Diet 5	46.80 ± 3.18	47.78 ± 3.01	0.89
Diet 6	86.53 ± 4.83	86.48 ± 3.25	0.26

Mean with the different letters in same row are significantly different at *p* ≤ 0.05.

The small loss of TC can be explained by the diet processing methods. The percentage losses of TC in both subgroups of diets, with and without antioxidant, are in the range of 2.07–5.21 and 2.26–4.96, respectively, and after the drying process they are from 3.03–7.82 and 2.67–8.47, respectively. 

The major causes of carotenoid destruction during processing and storing of dietary carotenoids is enzymatic or non-enzymatic oxidation. Thus, carotenoids are susceptible to the loss of provitamin A activity through oxidation during processing [14]. The reported retention values for astaxanthin in extruded diets range from 86% [15], depending on processing parameters. Similar stabilities were observed previously [16]. Therefore, total carotenoid stability in the final product in this study depended mostly on processing temperature. 

Diets that passed through the die surface would still contain 30–40% of moisture, so the drying process by hot air oven was needed in order for the moisture to evaporate. However, using very high temperature reduces the amount of nutrients in diets during the process. Using low temperatures would require longer periods, but the loss of nutrition in the feeds would be less. All parts of the diets require constant heat and air so that the diets would dry faster and contain the nutrition. A hot air oven was used for this process at 60 °C for 12 h. Haaland *et al*. [17] reported that the optimal temperature for drying pellets would leave the pellet mill at temperatures as high as 190 °F (87.8 °C). Hence, the temperature of the dryer in this study was safe for the loss of nutrition. 

This study found that in diets formulated as fish feed, carotenoid content remained quite stable. Pelleting process of the diets (with a pelleting machine: soft type pellet without steam) did not affect the carotenoid content of the diets.

### 2.2. Stability of Carotenoid Diets under Different Storage Temperatures

The dietary carotenoids were split into two smaller groups, with and without antioxidant. After the diets were stored under different storage temperatures, dietary carotenoids were stored in aluminum foil bags and the top sealed with a bag sealer. The bags were kept at different temperatures for eight weeks. Random samples were collected at 0, 4 and 8 weeks to analyze the quantities of TC in the diets (as shown in Table 3 and Table 4).

**Table 3:** Total carotenoids content in the experimental diets storage under room temperature during the trial period.

Experimental Diets	Total carotenoid (mg kg^−1^)	
	Without antioxidant	With antioxidant
**Week 0**		
Diet 1	5.54 ± 0.19	5.54 ± 0.19
Diet 2	25.56 ± 0.98	25.56 ± 0.98
Diet 3	23.67 ± 2.45	23.67 ± 2.45
Diet 4	25.04 ± 2.97	25.04 ± 2.97
Diet 5	45.85 ± 4.72	45.85 ± 4.72
Diet 6	84.97 ± 4.28	84.97 ± 4.28
**Week 4**		
Diet 1	5.46 ± 0.38	5.65 ± 0.42
Diet 2	24.04 ± 3.39	26.22 ± 5.01
Diet 3	22.88 ± 0.67	24.64 ± 3.33
Diet 4	23.83 ± 1.59	25.36 ± 1.55
Diet 5	43.70 ± 0.75	45.40 ± 0.81
Diet 6	82.85 ± 2.39	84.50 ± 2.24
**Week 8**		
Diet 1	5.28 ± 0.73	5.55 ± 0.10
Diet 2	23.27 ± 3.93	25.55 ± 2.36
Diet 3	21.83 ± 1.72	25.07 ± 1.07
Diet 4	23.17 ± 2.16	25.03 ± 0.72
Diet 5	42.45 ± 4.85	45.02 ± 2.33
Diet 6	80.74 ± 4.33	83.75 ± 1.25
*P*-value	0.0001	0.0001
Diet	0.0001	0.0001
Storage time	0.0597	0.9372
Diet*Storage time	0.9962	0.9997

**Table 4:** Total carotenoids content in the experimental diets storage under 4 °C during the trial period.

Experimental Diets	Total carotenoid (mg kg^−1^)	
	Without antioxidant	With antioxidant
**Week 0**		
Diet 1	5.54 ± 0.19	5.54 ± 0.19
Diet 2	25.56 ± 0.98	25.56 ± 0.98
Diet 3	23.67 ± 2.45	23.67 ± 2.45
Diet 4	25.04 ± 2.97	25.04 ± 2.97
Diet 5	45.85 ± 4.72	45.85 ± 4.72
Diet 6	84.97 ± 4.28	84.97 ± 4.28
**Week 4**		
Diet 1	5.46 ± 0.38	5.65 ± 0.42
Diet 2	24.04 ± 3.39	26.22 ± 5.01
Diet 3	22.88 ± 0.67	24.64 ± 3.33
Diet 4	23.83 ± 1.59	25.36 ± 1.55
Diet 5	43.70 ± 0.75	45.40 ± 0.81
Diet 6	82.85 ± 2.39	84.50 ± 2.24
**Week 8**		
Diet 1	5.28 ± 0.73	5.55 ± 0.10
Diet 2	23.27 ± 3.93	25.55 ± 2.36
Diet 3	21.83 ± 1.72	25.07 ± 1.07
Diet 4	23.17 ± 2.16	25.03 ± 0.72
Diet 5	42.45 ± 4.85	45.02 ± 2.33
Diet 6	80.74 ± 4.33	83.75 ± 1.25
*P*-value	0.0001	0.0001
Diet	0.0001	0.0001
Storage time	0.0597	0.9372
Diet*Storage time	0.9962	0.9997

There is no effect in the change of TC quantities in diets from all groups that were kept at different temperatures during the trial period (Table 5).

**Table 5:** Comparing total carotenoids content in the experimental diets with and without antioxidant at 26–28 °C and 4 °C during 8 weeks.

Experimental diets	Total carotenoid (mg kg^−1^)		
	Without antioxidant	Antioxidant	*P*-value
**Room temperature**			
Diet 1	5.43 ± 0.43	5.58 ± 0.24	0.35
Diet 2	24.29 ± 2.76	25.78 ± 2.78	0.19
Diet 3	22.79 ± 1.61	24.46 ± 2.28	0.75
Diet 4	24.01 ± 2.24	25.14 ± 1.74	0.08
Diet 5	43.99 ± 3.43	45.43 ± 2.51	0.11
Diet 6	82.85 ± 3.67	84.41 ± 2.59	0.16
**Cool temperature (4 °C)**			
Diet 1	5.47 ± 0.16	5.66 ± 0.14	0.42
Diet 2	25.25 ± 2.69	26.45 ± 2.21	0.55
Diet 3	23.36 ± 1.92	25.60 ± 0.94	0.47
Diet 4	24.56 ± 1.62	25.67 ± 2.18	0.80
Diet 5	44.81 ± 3.39	46.41 ± 2.34	0.19
Diet 6	83.74 ± 2.31	85.42 ± 1.77	0.35

Mean with the different letters in same row are significantly different at *p* ≤ 0.05.

TC decrease in diets without antioxidant was more than those with antioxidant. Stored longer at room temperature, 4.79–8.97 and 1.30–2.58 of TC are lost during the 8-week period. The diets stored at 4 °C had 2.11–3.8 and 1.09–3.86 TC loss, respectively. It can be concluded that keeping the carotenoid diets (with and without antioxidant) at 4 °C is more effective in terms of slowing down the loss of TC quantities.

Heat, light and oxygen might have been the agents that most contributed to total carotenoid degradation, and this degradation was continuous even during storage [18]. Total carotenoid stability of all formulated carotenoid diets seemed to be affected predominantly by storage temperature which is in accordance with many other observations [19,20,21,22]. The data from this study indicated that the best storage temperature for formulated carotenoids diet was 4 °C.

## 3. Experimental

### 3.1. Diets

The various carotenoids were premixed with rice bran and included in the dry feed mixture at planned concentrations. All diets were split into two groups, of which one contained BHT (an antioxidant) at 250 ppm and the other did not contain BHT. The ingredients and proximate compositions of the experimental carotenoid diets are shown in Table 6. The diets were produced with a HOBRAT mincer: model 4730, designed 3 H.P. The dry mix was pelletized in a single screw (soft-type pellet without steam) with a length of 30 cm passed through a modifier (ratio diameter of input: output, 3:1 mm). The feed was processed to obtain a feed particle size of 2–3 mm. The feed was dried with hot air at 60 °C in an oven for approximately 12 h. Before bagging, the feed was cooled by being left at room temperature (26–28 °C) for 2 h, and then was equally divided into aluminum foil bags. 

**Table 6:** Ingredients and proximate composition of the experimental carotenoid diets.

Ingredient (Kg)	Experimental diets					
	1	2	3	4	5	6
Fish meal	30	30	30	30	30	30
Soybean meal	24	24	24	24	24	24
Rice bran	24	24	24	24	24	24
Tapioca starch	5	5	5	5	5	5
Wheat Flour	5	5	5	5	5	5
Fish oil	2	2	2	2	2	2
Alpha-starch	5	5	5	5	5	5
Dicalcuimphosphate	1	1	1	1	1	1
Premix	2	2	2	2	2	2
Astaxanthin	-	✓	-	-	-	-
Lutein	-	-	✓	-	✓	✓
β-carotene	-	-	-	✓	✓	✓
Lecithin	2	2	2	2	2	2
Total	100	100	100	100	100	100
**Proximate composition by analysis (% dry weight on basis)**						
Protein	29.54 ± 2.05					
Fat	5.05 ± 0.29					
Fiber	4.81 ± 0.06					
Moist	6.40 ± 0.70					
Ash	9.36 ± 0.96					
**Carotenoids compositions by analysis (mgkg^−1^ dry weight on basis)**						
Total carotenoids	4.45 ± 0.46	29.51 ± 2.12	27.73 ± 2.73	27.01 ± 3.65	57.43 ± 3.36	109.39 ± 7.84
Astaxanthin	ND.	25.79 ± 0.81	ND.	ND.	ND.	ND.
Lutein	ND.	ND.	23.35 ± 2.92	ND.	25.53 ± 5.71	46.31 ± 5.71
β-carotene	ND.	ND.	ND.	24.14 ± 1.51	28.57 ± 0.57	59.54 ± 1.14

Experimental diets were designed to contain carotenoids as follows:
Diet 1: Control diet with low total carotenoid of at least 5 ppm. Diet 2: Diet supplemented with astaxanthin at 25 ppm. Diet 3: Diet supplemented with lutein at 25 ppm. Diet 4: Diet supplemented with β-carotene 25 ppm. Diet 5: Diet combined with lutein and β-carotene at 25:25 ppm. Diet 6: Diet combined with lutein and β-carotene at 50:50 ppm. Diets were maintained for 2 months under the following conditions: A: Stored at room temperature of 26–28 °C. B: Stored in refrigerator at 4 °C.

### 3.2. Sampling and Analyses

**Figure 1:**
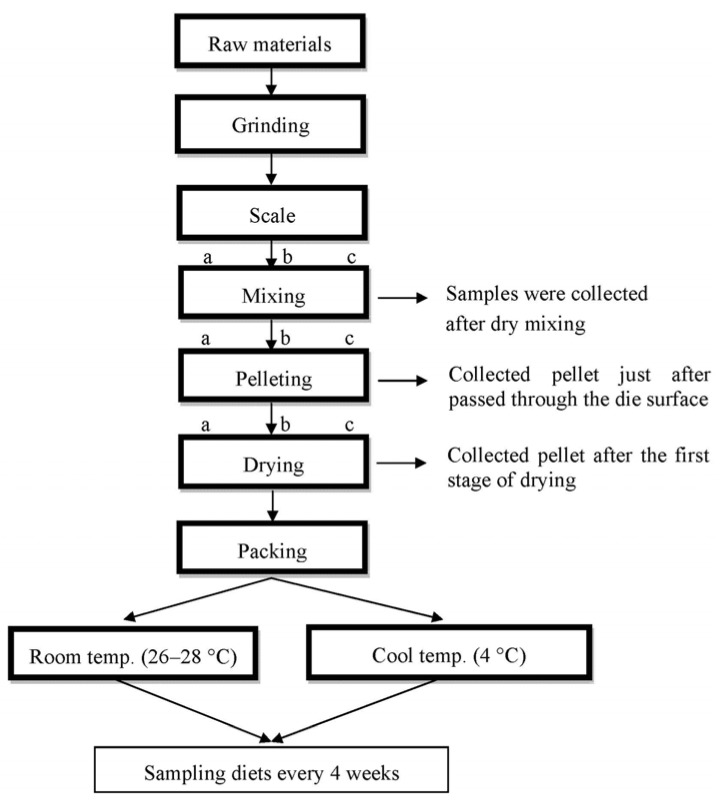
Scheme illustrating the feed processing and sampling diets in the study; samples were collected three locations (a, b and c).

Three samples (approximately 50 g) of each diet were taken from the dry mix, after being processed and dried, respectively. Three areas in the mash of diets were randomized for sampling collection. The samples were kept immediately in a freezer at −10 °C. The dry matter of all samples was analyzed for total carotenoid (TC) contents. The mixer was divided into ree locations, and two samples were collected from each location for analysis. Samples of processed feed were collected once stable operating conditions were reached. During processing, samples for analyses were taken of the feed just after passed through the die surface and of the pellet after the first stage of drying. Three samples were taken and pooled and three pooled samples were taken per treatment per sampling location (n = 3 for each sampling location). Feed processing and sampling test diets have the following steps are presented in Figure 1.

### 3.3. Total Carotenoids Determination

Three replicated samples of all diets from unit operations were extracted with cool acetone, together with added BHT (250 ppm) as antioxidant, until the samples lost their color. After that, 5 mL petroleum ether was added, mixed and water added in a separating funnel, then mixed with a careful swirling until the two phases separated. In this study, only the hyperphase was collected, and then, the total carotenoid quantity was determined. Total carotenoid (TC) content in all diets was determined after extraction. Carotenoids amount was calculated spectroscopically by UV-Vis at 450 nm with A^1%^ = 2,500 [23].

### 3.4. Statistical Analysis

All data were subjected to a factorial consisting of two factors to investigate the interaction between dietary carotenoids and processing methods (dry mix, after being processed and dryer) for study of feed processing, and the interaction between dietary carotenoids and storage time for studying carotenoid stability under different storage temperatures during storage. The mean value and the standard deviation (S.D.) were calculated from the results. Two-way ANOVA was applied for the comparison of the mean values where *p* ≤ 0.05 was regarded as significant.

## 4. Conclusions

This study found that TC levels in all diets remained quite stable without significant loss during feed processing. The pelleting process of the diets (with a pelleting machine: soft-type pellet without steam) did not affect the carotenoid content of the diets. Refrigerated storage is one option for slowing down TC loss; the other possibility is to add antioxidants. However, added antioxidants would be convenient with energy saved before the feed was processed. Therefore, low temperature and antioxidant addition effectively prevent TC loss of dietary carotenoids.
